# Down-Regulation of Colonic ACE2 Expression in Patients With Inflammatory Bowel Disease Responding to Anti-TNF Therapy: Implications for COVID-19

**DOI:** 10.3389/fmed.2020.613475

**Published:** 2021-01-12

**Authors:** Xiao-Zhi Li, Yun Qiu, Louisa Jeffery, Fen Liu, Rui Feng, Jin-Shen He, Jin-Yu Tan, Zi-Yin Ye, Si-Nan Lin, Subrata Ghosh, Marietta Iacucci, Min-Hu Chen, Ren Mao

**Affiliations:** ^1^Department of Gastroenterology, The First Affiliated Hospital of Sun Yat-sen University, Guangzhou, China; ^2^Institute of Immunology and Immunotherapy, University of Birmingham, Birmingham, United Kingdom; ^3^National Institute for Health Research Biomedical Research Center, University of Birmingham and University Hospitals Birmingham NHS Foundation Trust, Birmingham, United Kingdom; ^4^Department of Pathology, The First Affiliated Hospital of Sun Yat-sen University, Guangzhou, China

**Keywords:** COVID-19, ACE2, inflammatory bowel disease, intestine, anti-TNFα

## Abstract

**Background and Aims:** Angiotensin-converting enzyme II (ACE2) is the key molecule for understanding the pathophysiology of COVID-19. The risk of COVID-19 and impact of immunosuppressive treatment on disease course in patients with inflammatory bowel disease (IBD) remain controversial. We aimed to determine the change of intestinal ACE2 expression before and after biologics treatment including anti-tumor necrosis factor α (anti-TNFα), anti-integrin, and anti-interleukin (IL)12/23 in IBD patients.

**Methods:** We analyzed the ACE2 expression through the public database of paired intestinal biopsies from IBD patients before and after biologic therapy. Change of ACE2 RNA and protein expression were validated in two independent cohorts (Birmingham cohort and Guangzhou cohort). The correlation between ACE2 expression and disease activity was also analyzed.

**Results:** Mining information from the GEO database showed that compared with healthy control, intestinal ACE2 expression was downregulated in ileum of CD patients, while upregulated in colon of both CD and UC patients. Colonic ACE2 RNA expression was decreased significantly in patients responding to anti-TNFα but not anti-integrin and anti-IL12/23, which was validated in the Birmingham cohort. Using the Guangzhou cohort including 53 patients matched by pre- and post-anti-TNFα therapy, colonic ACE2 protein expression was significantly downregulated after anti-TNFα treatment in responders (*P* < 0.001) rather than non-responders. Colonic ACE2 expression was significantly higher in patients with severe histologically active disease compared with those with moderate (*P* < 0.0001) and mild (*P* = 0.0002) histologically active disease.

**Conclusion:** Intestinal inflammation influences the expression of intestinal ACE2 in IBD patients, with different alterations in the ileum and colon. Colonic ACE2 expression was downregulated after anti-TNFα therapy in IBD patients responding to treatment. This might provide new clues regarding the risk of SARS-CoV-2 infection and the potential benefit of sustaining anti-TNFα treatment in patients with IBD.

## Introduction

The outbreak of coronavirus disease 2019 (COVID-19), caused by severe acute respiratory syndrome coronavirus 2 (SARS-CoV-2), has widely spread around the world. Angiotensin-converting enzyme II (ACE2) has emerged as a key molecule in the pathophysiology of COVID-19 (1). ACE2 is expressed in the respiratory tract as well as gastrointestinal tract (2, 3). Emerging data (4–7) showed that the SARS-CoV-2 may actively infect and replicate in the human gut enterocytes or organoids and might be transmitted via fecal-oral transmission.

Given the use of immunosuppressive agents as well as malnutrition status, patients with inflammatory bowel disease (IBD) are generally at increased risk of infection. Unexpectedly, current epidemiology of SARS-CoV-2 infection in IBD patients, including published reports from China (8), Spain (9), Italy (10, 11), and the global data from the SECURE-IBD registry (https://covidibd.org/) (12), did not support a higher risk of COVID-19 in IBD patients compared to that in the general population.

Similar immune signatures in IBD and COVID-19 indicate that medications of IBD may play a potential role in the treatment of COVID-19. Some studies supported that infliximab downregulated ACE2 expression in colon tissue of IBD (13, 14). Another recent study has showed that biologics and steroids are linked to the significantly lower expression of ACE2 in intestinal lamina propria CD11b-enriched cells (15). However, to our knowledge, few studies have investigated the influence of biologics on ACE2 expression in gut enterocytes which are directly exposed to the virus. Our study identified the ACE2 expression with the public database and then validated RNA and protein expression using clinical samples from two independent cohorts including China and the UK aiming to determine the alter expression of intestinal ACE2 especially in enterocytes before and after biologic therapy including anti-tumor necrosis factor α (anti-TNFα), anti-integrin and anti-interleukin (IL) 12/23 in IBD patients.

## Methods

### Transcriptomic Change of Intestinal ACE2 Pre- and Post-biologic Therapy From the Gene Expression Omnibus (GEO) Database

We searched the gene expression data sets regarding biologics in IBD in the GEO database (http://www.ncbi.nlm.nih.gov/geo/) with the key words “Inflammatory bowel disease/Crohn's disease/Ulcerative colitis/IBD/CD/UC,” “intestine/tissue” and “Homo sapiens.” The inclusion criteria from the data sets were: (1) intestinal tissue from patients with IBD; (2) paired samples were collected before and after various biologics and small molecule inhibitors therapy including but not limited to anti-TNFα, anti-integrin, and anti-IL12/23; (3) therapeutic efficacy (i.e., response or not) of each patient was described; (4) the number of samples per group was not <12 (16). We extracted the expression value of ACE2 and used two-class paired or unpaired analyses according to experimental design. Value distributions were evaluated.

### Validation of Intestinal ACE2 RNA Expression Change by Real-Time Quantitative Polymerase Chain Reaction (RT-qPCR) Pre- and Post-anti-TNFα Therapy

This validation was conducted in a cohort at the University of Birmingham, UK. Colonoscopy-confirmed active UC and CD patients were recruited prior to initiation of anti-TNFα (Infliximab [IFX] or adalimumab [ADA]) therapy. Institutional research ethics approval was obtained for the study and all patients had signed informed consent.

Colonoscopic biopsies were taken from the inflamed segments of IBD patients (UC and CD) before and 12–16 weeks after starting treatment with biologics. The endoscopic response was judged by Mayo endoscopic score 0–1 or Simple Endoscopic Score-CD decrease of 50% or greater at week 12–16 compared to baseline (17). Biopsies were transferred immediately to “RNA later” upon collection and stored at 4°C prior to lysis and gentleMACS homogenization (Miltenyi Biotec) followed by RNeasy on-column RNA extraction and purification (Qiagen). RNA was quantified by Qubit (Life Technologies) and 1.5 μg reverse transcribed using iScript reagents (Bio-Rad). Expression of ACE2 receptor relative to 18SrRNA was measured by qPCR using Taqman reverse transcription gene-assays (18SrRNA: 4319413E. ACE2: Hs01085333_m1) (Life Technologies). Reactions were performed in triplicate as singleplex assays and expression of ACE2 relative to 18SrRNA calculated by 10^6^(2^−dCt^). The expression change between pre- and post-treatment was tested.

### Validation of Intestinal ACE2 Protein Expression Change Using Immunohistochemistry (IHC) Assays Pre- and Post-anti-TNFα Treatment

To determine the protein expression of ACE2 in the intestinal epithelial cells, patients with CD receiving anti-TNFα treatment were included from the First Affiliated Hospital of Sun Yat-sen University, Guangzhou, China. All patients underwent colonoscopy by gastroenterologists with more than 5 years of experience in IBD before and 12–14 weeks after anti-TNFα treatment, and all biopsies were taken from inflamed gut segments. Institutional research ethics approval was obtained for the study.

IHC was performed using paraffin-embedded tissues from intestinal mucosal biopsies obtained during endoscopy from the IBD patients mentioned above. Tissue sections were deparaffinized in xylene and hydrated through a graded series of alcohol to tap water. Antigen was retrieved in citrate buffer for 20 min and cooled to room temperature. The sections were incubated with 3% H_2_O_2_ in distilled water for 15 min. After being rinsed three times with phosphate-buffered saline (PBS), the sections were incubated with rabbit polyclonal IgG primary antibodies against ACE2 (1:500 dilution, ab15348; Abcam; USA) overnight at 4°C. Then, the sections were incubated with the secondary antibody (1:50000, ab205718; Abcam; USA) for 30 min at room temperature, followed by two times 5 min washing with PBS. Finally, the sections were stained with hematoxylin. The protein expression of ACE2 was evaluated in a random and blinded fashion and was assigned an IHC score, which was based on the approximate percentage of positively stained cells over overall intestinal epithelial cells (ranging from 0 to 100%), as described in previously published methods (18).

### Assessment of Disease Activity and Definition of Outcome

The disease activity of the CD patients from the China cohort was analyzed using endoscopic and histologic assessment. The Crohn's Disease Endoscopic Index of Severity (CDEIS) score was used for endoscopic scoring. Consistent with the STRIDE guidelines (17), a decrease by >5 or at least 50% from baseline in CDEIS demonstrates endoscopic response. A semiquantitative evaluation for endoscopic disease activity was given as follows: CDEIS <3 suggested inactive, 3–8 mildly active, 9–12 moderately active, and >12 severely active (19).

Histological disease activity was assessed by a blinded IBD experienced pathologist in random order. The modified Global Histologic Disease Activity Score (mGHAS) (20, 21) was used and the histologic response was defined as modified GHAS ≤4 in those patients with baseline score >4. A semiquantitative evaluation for histological disease activity was given as follows: inactive, 0; mildly active, 1–5; moderately active, 6 −10; and severely active, 11–14.

### Statistical Analysis

The original expression data was collected and then plotted by GraphPad Prism 8 (GraphPad Software, La Jolla, CA). All statistical analyses were performed IBM SPSS Statistics 25.0 software package (IBM, Armonk, NY, USA). Continuous variables were summarized as medians and interquartile ranges (IQRs). The Student's *t*-test or analysis of variance (ANOVA) test was used for parametric tests, while the Wilcoxon signed-rank test or Kruskal-Wallis test was performed for non-parametric tests. The Spearman correlation was used to evaluate the relationship of IHC score and endoscopic or histological disease activity score. All statistical testing was two-sided, and *P* < 0.05 was considered significant and indicated as follows: ns, not significant; ^*^P ≤ 0.05; ^**^P ≤ 0.01; ^***^P ≤ 0.001; ^****^P ≤ 0.0001.

## Results

### Intestinal ACE2 Expression in GEO Database

Five GEO datasets [GSE16879 (22), GSE23597 (23), GSE92415 (24), GSE73661 (25), and GSE112366 (26)] were included in the final analysis. Detailed information for datasets included was summarized in Table 1. Compared with healthy control, intestinal ACE2 expression was downregulated in ileum of CD patients (GSE16879), while upregulated in colon of both CD and UC patients (GSE16879, GSE92415, and GSE73661), significantly.

**Table 1:** Summary of included GEO datasets.

**Patients Cohort**	**Healthy Control**	**IBD Type**				**Response or not**		**Matched Comparison (Yes/No)**	**Sample Source**	**Study Time Point^c^**	**Definition of Outcome^d^**	**Treatment**	**GEO Dataset**
		**CD^a^**			**UC**	**Responders Pair^b^**	**Non-responders Pair**						
		**Ileal CD (L1)**	**Colonic CD (L2)**	**Ileocolonic CD (L3)**									
Leuven, Belgium (20)	12	18	19	0	24	28L1: 8L2: 12UC: 8	33 L1: 10 L2: 7 UC: 16	Yes	ileum (L1), colon (L2)	weeks 0, 4/6	Endoscopic and Histological score	IFX (5mg/kg)	GSE16879
ACT1 study (21)	0	0	0	0	48	49IFX/PBO w0-8: 18/3IFX/PBO w0-30: 14/4IFX/PBO w0-8-30: 9/1	28 IFX/PBO w0-8: 7/5 IFX/PBO w0-30: 5/6 IFX/PBO w0-8-30: 3/2	Yes	colon	weeks 0, 8, 30	Endoscopic score	IFX (5/10 mg/kg) PBO	GSE23597
PURSUIT-SC study (22)	21	0	0	0	162	82 (unpaired)GLM w0/6: 32/29PBO w0/6: 11/10	80 (unpaired) GLM w0/6: 27/21 PBO w0/6: 17/15	No	colon	weeks 0, 6	Endoscopic score	GLM	GSE92415
GEMINI study (23)	12	0	0	0	67	29IFX w0-4/6: 8VDZ w0-6: 6VDZ w0-12: 5VDZ w0-52: 10	51 IFX w0-4/6: 15 VDZ w0-6: 21 VDZ w0-12: 10 VDZ w0-52: 5	Yes	colon	IFX weeks 0, 4/6VDZ weeks 0, 6, 12, 52	Endoscopic score	IFX, VDZ, PBO	GSE73661
UNITI study (24)	26	26	18	54	0	56w0-8: 35w0-44: 21	42 w0-8: 29 w0-44: 13	Yes	ileum	weeks 0, 8, 44	CDAI score	UST	GSE112366

a Disease subtypes are classified according to Montreal classification;

b *Responders/non-responders pair, one pair means that the patient has biopsy samples before and after treatment*;

c *Study time point, time to definite response status*;

d *Definition of outcome, Method to definite response/non-response after treatment*.

*IBD, inflammatory bowel disease; CD, Crohn's disease; UC, ulcerative colitis; IFX, infliximab; GLM, golimumab; VDZ, vedolizumab; UST, ustekinumab; PBO, placebo; CDAI, Crohn's Disease Activity Index*.

**Figure 1:**
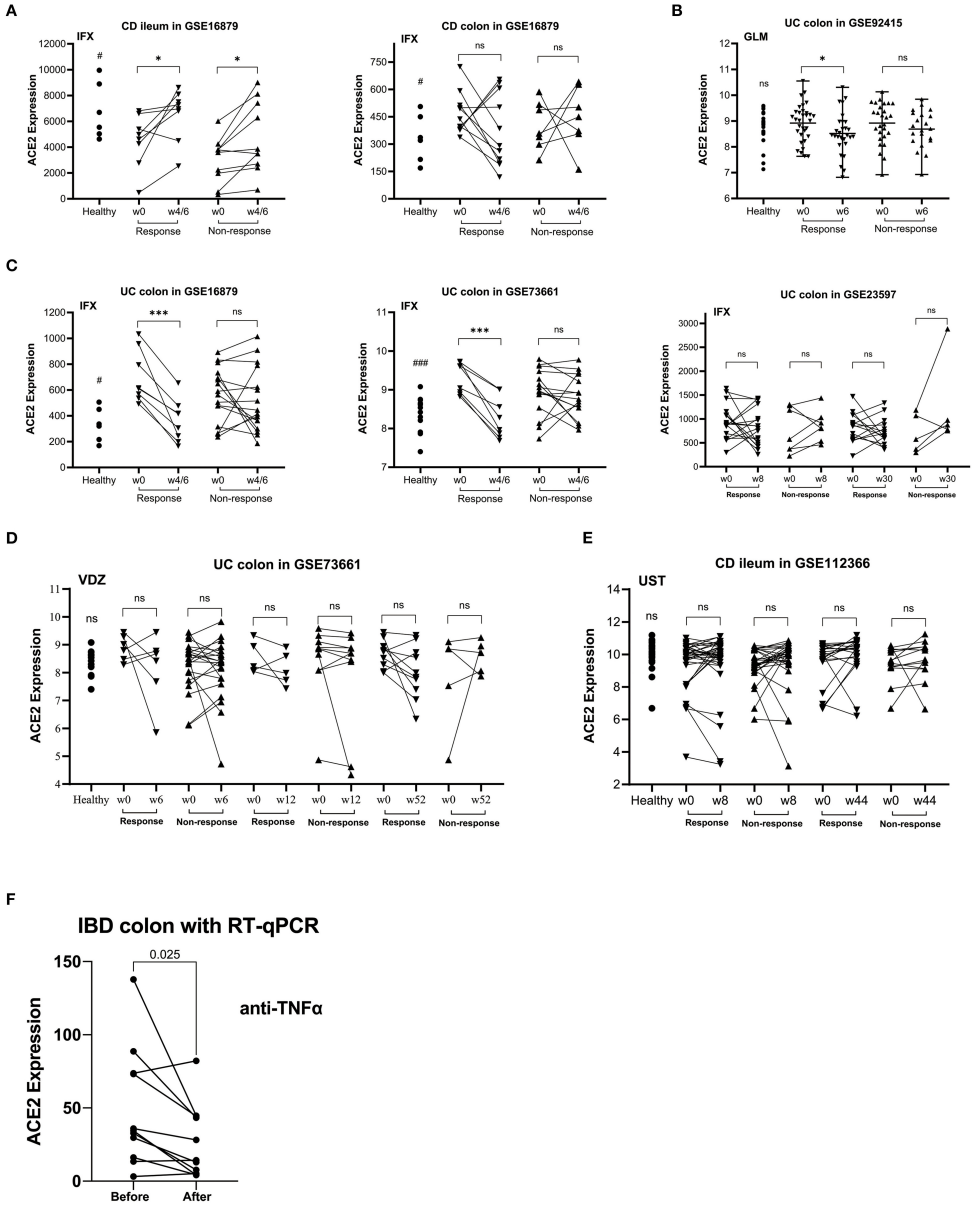
The relative ACE2 mRNA expression level of intestinal mucosal biopsy specimens before and after biologic therapy with anti-TNFα (infliximab/IFX, **A,C**; golimumab/GLM, **B**), vedolizumab/VDZ **(D)** or ustekinumab/UST **(E)** in patients with CD **(A,E)** or UC **(B–D)** from GEO data sets. **(F)** RT-qPCR data of the intestinal mucosal ACE2 expression in IBD responders before and after anti-TNFα therapy. In the matched comparison **(A,C–F)**, lines between two samples represent the change in ACE2 expression before and after treatment for one patient. In the unpaired comparison **(B)**, mean and range are shown in the scatterplot. There was not statistical difference between the IBD patients before and after therapy. ns, not significant; *P < 0.05; ***P < 0.001 (in patients after vs. before therapy). ^#^*P* < 0.05; ^*###*^*P* < 0.001 (in healthy controls vs. patients before therapy).

As shown in Figures 1A–C, ACE2 expression in colon tissue was decreased significantly in patients responding to anti-TNFα (except GSE23597). On the contrary, ACE2 in ileum tissue was upregulated significantly in CD patients using anti-TNFα regardless of the response status (GSE16879). Intestinal ACE2 expression did not decrease after VDZ or UST treatment (Figures 1D,E).

### Colonic ACE2 RNA Expression Was Downregulated in IBD Patients Responding to anti-TNFα

In the UK cohort, we studied 24 IBD patients (11 CD, 13 UC) initiating biologic therapies (CD 8 ADA, 2 IFX, 1 UST; UC 5 ADA, 4 IFX, 4 VDZ). The baseline characteristics of patients are shown in Table 2. Figure 1F shows a statistically significant decrease in colonic expression of ACE2 in responders (*n* = 11) to anti-TNFα (*P* = 0.0250). Non-responders to anti-TNFα (4 UC, 1 CD) did not exhibit any significant decrease in ACE2 expression. Patients treated with VDZ did not show any significant decrease in ACE2 expression.

**Table 2:** Baseline characteristics of patients with inflammatory bowel disease enrolled in validation cohorts.

**Characteristics**	**Total Patients (China cohort)**	**Total Patients (UK cohort)**
No. of patients	66	24
Male, *N* (%)	43 (65.2%)	12 (50%)
Age at time of collection (years)		
Mean (SD)	23.4 (8.9)	42.0 (12.6)
Range	10–46	24–63
Duration of disease (months)		
Mean	44.7 (55.8)	121.0 (106.0)
Range	1–366	6–372
Crohn's Disease (CD), *N*	66	11
Location, *N*		
L1, Ileal	5	4
L2, Colonic	3	5
L3, Ileocolonic	58	2
Disease behavior, *N*		
B1, Non-stricturing, non-penetrating	39	6
B2, Stricturing	19	3
B3, Penetrating	8	2
Ulcerative Colitis (UC), *N*	0	13
Proctitis (E1)	0	1
Left sided (E2)	0	5
Extensive (E3)	0	7
Biologics commenced, *N*		
Anti-TNF	66 (all CD)	19 (10 CD, 9 UC)
Vedolizumab	0	4 (all UC)
Ustekinumab	0	1 (CD)

### Colonic ACE2 Protein Expression Was Downregulated in CD Patients Responding to anti-TNFα

**Figure 2:**
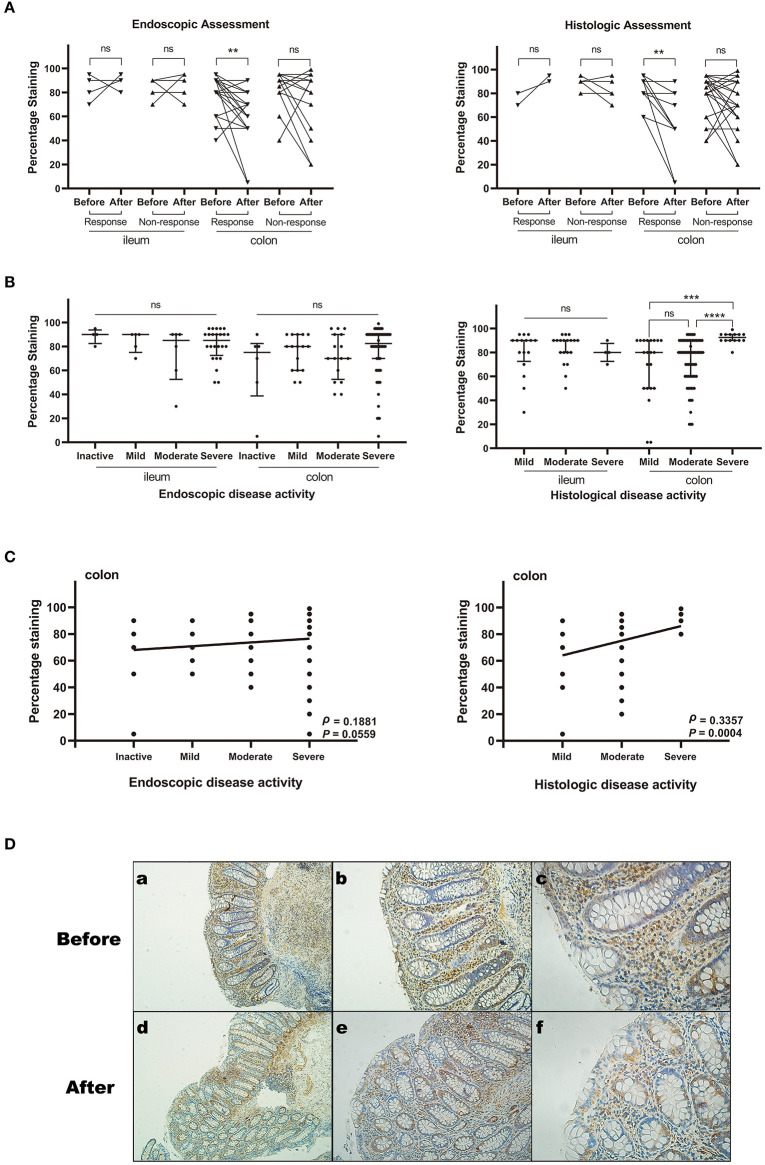
The relative ACE2 protein expression in intestinal mucosal biopsy specimens from patients with CD by Immunohistochemistry assays. The expression level was measured by percentage of positively stained cells. The sample sizes of each group are shown in Supplementary Table 1. **(A)** ACE2 expression before and after anti-TNFα treatment (matched comparison). **(B)** ACE2 expression among different disease activity groups defined by endoscopic and histological assessment. Median and interquartile range are shown in the scatterplot. **(C)** Spearman rank correlation analysis between ACE2 expression of colonic epithelial cells and endoscopic or histological disease activity. **(D)** Representative images (immunohistochemical staining for ACE2) of the colonic biopsy specimens before and after anti-TNFα treatment. (a–c) Biopsy before treatment. The histological score is 6. The percentage staining of ACE2 in colonic epithelial cells is 80%. (d–f) Biopsy after treatment. The histological score is 1. The percentage staining of ACE2 in colonic epithelial cells is 20%. All scale bars are 100 μm. ns, not significant; **P < 0.01; ***P < 0.001, ****P < 0.0001.

In the China cohort, we included 66 CD patients for IHC validation and found 53 patients matched by pre- and post-anti-TNFα therapy (Supplementary Table 1). The baseline characteristics of patients are shown in Table 2. As demonstrated in Figure 2A, in both endoscopic and histologic assessments, ACE2 expression was significantly downregulated in colonic biopsy after anti-TNFα treatment in responders (*P* < 0.001) rather than non-responders (the representative IHC images are shown in Figure 2D). Besides, ACE2 protein expression in ileum increased after anti-TNFα treatment in responders (*n* = 3). There was no difference in ACE2 protein expression in colon or ileum of non-responders.

### Colonic ACE2 Protein Expression Positively Correlated With Disease Activity

We studied 147 specimens with different disease activity from total CD patients of the China cohort. Colonic ACE2 expression was significantly higher in patients with severe histologically active disease compared with those with moderate (*P* < 0.0001) and mild (*P* = 0.0002) histologically active disease. Ileal ACE2 expression was comparable among different disease activity groups. When stratifying the disease activity by endoscopic score, no significant difference existed among disease activity groups (Figure 2B).

As is shown in Figure 2C, ACE2 expression positively but weakly correlated with histological disease activity (ρ = 0.3357, 95% confidence interval [CI] 0.1502 to 0.4983, *P* = 0.0004) and endoscopic disease activity (ρ = 0.1881, 95% CI −0.0105–0.3723, *P* = 0.0559) in colon, while no correlation existed between ileal ACE2 expression and histological or endoscopic disease activity.

## Discussion

There are controversies about the risk of SARS-CoV-2 infection in patients with IBD (27). It was reported that the soluble form of ACE2, which acts as a competitive binding partner for SARS-CoV-2, is up-regulated in the peripheral blood of IBD patients and then limits SARS-CoV-2 infection (28, 29). Several studies showed that IBD medications especially biologics could regulate the intestinal ACE2 expression of IBD (13–15). However, few studies have directly investigated the influence of biologics on ACE2 expression in gut enterocytes which are directly exposed to the virus. The two recent landmark studies (6, 7) have confirmed that SARS-CoV-2 could productively infect human gut enterocytes and intestinal organoids. In our study, we found that IBD patients had a higher expression of ACE2 in colon tissue while lower in ileum tissue vs. healthy control, which was consistent with the published data (13). Additionally, our result showed that the expression of colonic epithelial ACE2 was downregulated in IBD patients responding to anti-TNFα therapy, using GEO data analysis and then validated with qPCR and IHC assays. These results might provide new evidence and knowledge to the risk of SARS-CoV-2 infection in patients with IBD using different medications and the potential role of anti-TNFα in the treatment of COVID 19.

Our study demonstrated that intestinal epithelial ACE2 expression increased with more severe disease activity, which may be due to the higher inflammatory cytokines. Previous studies (30, 31) showed that ACE2 is increased in human bronchial epithelial cells infected by SARS-CoV-2 as a response to inflammatory cytokine stimulation including interferon (IFN)-γ. Several inflammatory cytokines like IFN-γ, TNFα, IL-1, and IL-6 could be upregulated in active IBD patients (28). In addition, ACE2 expression was downregulated after anti-TNFα therapy only in responders rather than non-responders. In the registered IBD patients with COVID-19 from SECURE-IBD (12), there were 762 patients with anti-TNFα therapy alone, 651 (85%) of whom recovered without hospital admission and four patients died in total. On the contrary, 65% of 773 patients with treatment of sulfasalazine/mesalamine recovered without hospital admission and 37 patients died. These data indicated that IBD patients with anti-TNFα treatment might have a better outcome of COVID than other medications (32). The potential explanations may have three points: (1) anti-TNFα treatment downregulated IFN-γ which would induced the expression of ACE2 through downregulating IFN-γ (32); (2) anti-TNFα treatment also downregulated other proinflammatory cytokines in “TNF dependent cytokine cascade,” such as IL-1, IL-6 and IFN-γ which also play important roles in cytokine storm syndrome in COVID-19 (32); (3) anti-TNFα could induce a reduction in leucocyte trafficking due to reduction of adhesion molecules, vascular endothelial growth factor and chemokines in both IBD and COVID-19 (33, 34). Indeed, an urgent demand for clinical trials of anti-TNFα therapy for COVID-19 has been proposed recently (35). Moreover, the clinical trial of anti-TNFα in treating COVID-19 (ChiCTR2000030089) is ongoing. Future studies investigating the protective role of anti-TNFα for IBD or COVID 19 patients during the COVID-19 pandemic are warranted.

Except for the regulation of inflammatory cytokines, ACE2 may participate in intestinal stem cell proliferation, mucosal healing and crypt pathology in the pathogenesis of IBD. ACE2 plays an important role in the endothelial repair in acute lung injury (36) and the healing of gastric ulcers (37), potentially through reducing Angiotensin (Ang) II and increasing the production of Ang 1–7. A recent study (38) proposed that ACE2 contributed to the proliferation of intestinal stem cells and the maintenance of epithelial barrier function in DSS-induced colitis mice. ACE2-deficient mice developed increased intestinal epithelial injury associated with crypt damage compared to the wild-type mice (39). However, there have been debates on the role of ACE2 in IBD. It is also reported that an ACE2 inhibitor may have an anti-inflammatory effect in DSS-induced colitis mice (40). Considering the dual role of ACE2 in the development of colitis, it warrants further study.

In the current study, a significant difference of ACE2 expression was found in responders rather than non-responders to anti-TNFα in IBD patients with colonic involvement, which was validated with IHC assays of CD patients in China cohort (Figure 2A). There are two patients with significant changes of ACE2, whose endoscopic and histological scores post-treatment are both close to zero. It demonstrated that the ACE2 may play an important role in the anti-TNFα mediated anti-inflammatory pathways in colonic CD. The difference was still statistically significant when taking out these two patients. Anti-TNFα is the mainstay of CD treatment. Nonetheless, around one-third of CD patients experience a loss of response (41). Besides, ACE2 or renin-angiotensin system (RAS) has been demonstrated to influence the inflammation and fibrosis in IBD (18). Thus, whether ACE2 or RAS could help for predicting response to anti-TNFα treatment deserves more research. It is unclear whether the concomitant medication influence ACE2 expression. In the present study, of 53 patients with intestinal biopsies pre- and post-anti-TNF therapy, none of them were on concomitant steroid use, and only one patient was on recent methylprednisolone use before anti-TNF therapy. There were 45 patients who received combination therapy of anti-TNF with azathioprine (Supplementary Table 2), most of whom had treatment failure of azathioprine before accelerating anti-TNF therapy. We further performed a subgroup analysis of patients on anti-TNF and azathioprine therapy and came to the same conclusion that colonic ACE2 was decreased significantly in patients responding to anti-TNFα (endoscopic response, *P* = 0.0096; histologic response, *P* = 0.0039). In recent studies (14, 32), international data from SECURE-IBD highlighted the association of corticosteroids with adverse COVID-19 outcomes and the probable safety of anti-TNF. The association between monotherapy or combination therapies and the risk of COVID-19 has been explored in some observational studies (14, 32, 42). Our study used paired samples before and after anti-TNF therapy, which could minimize the inter-individual differences such as concomitant medication. However, some confounding factors are inevitable in our current retrospective study. Further prospective well-designed studies are needed to validate our results.

Our study also showed that anti-TNFα could downregulate the ACE2 expression level in colon of patients with IBD rather than in ileum. ACE2 in ileum tissue was upregulated significantly in CD patients using anti-TNFα regardless of the response status (GSE16879, Figure 1A), which demonstrated that anti-TNFα may not influence the ileal ACE2 expression. Numerous previous evidence (43) supported that colonic CD is a different phenotype from ileal CD at the level of genetics, macroscopic, cellular immunology, microbiota, and treatment. It is worth mentioning that isolated ileal disease location has been observed to be a negative predictor of responses to anti-TNFα therapy in several cohort studies and there was no significant difference in efficacy of VDZ treatment in different locations (44). Therefore, we speculated that the RAS may be an important factor in the TNF-pathway of colonic CD and UC.

There was a positive correlation between epithelial ACE2 expression and disease activity, and the association was stronger using histological score compared to endoscopic score. Endoscopic and clinical measurements are predominately used to determine response to therapy in IBD. There has been growing interest in using histological score as measuring disease activity and treatment outcome. Previous studies (45) have shown that endoscopic assessment and clinical measures may not adequately reflect disease activity, whereas histologic measurement is more sensitive to detect disease activity and predict response to therapy.

One strength of the present study was that we included the data of matched intestinal mucosal biopsies from IBD patients before and after biologic therapy, so participant variables (i.e., individual differences) are reduced. Besides, we provided three sets of data to support our ACE2 expression changes after biologics use especially the down-regulation after anti-TNFα treatment and validated in IBD cohorts from different countries. More importantly, we not only assessed the disease activity and response by endoscopic score but also histological score which was better to illustrate the association between intestinal epithelial ACE2 expression and inflammatory activity.

Several certain limitations also existed. Firstly, because of the inconvenience of collecting biopsies from patients during the COVID19 pandemic, the validation of ACE2 mRNA and protein was conducted in two separate cohorts and a small amount of ileum tissue was included, which limited the assessment of the difference in ACE2 expression between terminal ileum and colon. Besides, we did not include samples for validation of ACE2 protein expression before and after VDZ/UST treatment, given these two biologics were not available in China before 2020. Finally, the validation of ACE2 protein expression did not included UC patients, because there were insufficient numbers of specimens of UC patients with anti-TNFα treatment to conduct statistical analysis. Further research is needed to confirm these findings.

In conclusion, our study showed that colonic ACE2 expression was downregulated after anti-TNFα therapy in IBD patients responding to treatment. This might provide new clues regarding the risk of SARS-CoV-2 infection and the potential benefit of maintaining anti-TNFα treatment in patients with IBD.

## Data Availability Statement

The original contributions generated for this study are included in the article/Supplementary Materials, further inquiries can be directed to the corresponding author/s.

## Ethics Statement

The studies involving human participants were reviewed and approved by Human Ethics Committee of the First Affiliated Hospital, Sun Yat-sen University. University of Birmingham Human Biomaterials Resource Centre. The patients/participants provided their written informed consent to participate in this study.

## Author Contributions

RM and M-HC conceived and supervised the overall study. X-ZL, MI, and RM wrote the manuscript. FL, RF, Z-YY, J-SH, J-YT, S-NL, SG, MI, M-HC, and RM critically revised the manuscript. X-ZL, YQ, and LJ performed the experiment and analyzed the data. All authors contributed to the article and approved the submitted version.

## Conflict of Interest

The authors declare that the research was conducted in the absence of any commercial or financial relationships that could be construed as a potential conflict of interest.
