# Supplementary material for: Down-Regulation of Colonic ACE2 Expression in Patients With Inflammatory Bowel Disease Responding to Anti-TNF Therapy: Implications for COVID-19
Source: Front Med (Lausanne). 2021 Jan 12;7:613475. doi: 10.3389/fmed.2020.613475 (PMC7835139; doi:10.3389/fmed.2020.613475)
Supplement: Supplementary file 1 [file Table_1.docx]

**Supplementary** Table 1. The number of specimens enrolled in IHC assays

|  | Definition of Outcome | | | | Total | Ileum | Colon |
| --- | --- | --- | --- | --- | --- | --- | --- |
| Matched pairs  (53 pairs) | Histologic score  （mGHAS） | | | Responders | 14 | 3 | 11 |
|  |  |  |  | Non-responders | 39 | 7 | 32 |
|  | Endoscopic score  (CDEIS) | | | Responders | 25 | 4 | 21 |
|  |  |  |  | Non-responders | 28 | 6 | 22 |
| Total Specimens  (147, including not-matched specimens) | | Histologic disease activity | mild | | 38 | 16 | 22 |
|  |  |  | moderate | | 91 | 19 | 72 |
|  |  |  | severe | | 18 | 4 | 14 |
|  |  | Endoscopic disease activity | inactive | | 10 | 4 | 6 |
|  |  |  | mild | | 21 | 5 | 16 |
|  |  |  | moderate | | 22 | 6 | 16 |
|  |  |  | severe | | 94 | 24 | 70 |

mGHAS, modified Global Histologic Disease Activity Score; CDEIS, Crohn’s Disease Endoscopic Index of Severity.

**Supplementary** Table 2. **Summary of** the concomitant medication in CD patients

| Definition of Outcome | | Anti-TNF Monotherapy | Concomitant Medication | | |
| --- | --- | --- | --- | --- | --- |
|  |  |  | AZA (azathioprine)^*^ | 6-MP (mercaptopurine) | SASP (Sulfasalazine) |
| Endoscopic assessment | Responders | 1 | 22 | 2 | 0 |
|  | Non-responders | 2 | 23 | 2 | 1 |
| Histologic assessment | Responders | 1 | 13 | 0 | 0 |
|  | Non-responders | 2 | 32 | 4 | 1 |

^*^, a subgroup analysis on colonic ACE2 expression pre- and post-anti-TNF combined with AZA therapy was performed. Endoscopic response, *P* = 0.0096; Histologic response, *P* = 0.0039.
